# From Classical Laboratory Parameters to Novel Biomarkers for the Diagnosis of Venous Thrombosis

**DOI:** 10.3390/ijms21061920

**Published:** 2020-03-11

**Authors:** Larisa Anghel, Radu Sascău, Rodica Radu, Cristian Stătescu

**Affiliations:** 1 Internal Medicine Department, “Grigore T. Popa” University of Medicine and Pharmacy, Iași 700503, Romania; larisa.anghel@umfiasi.ro (L.A.); rodiradu@hotmail.com (R.R.); cstatescu@gmail.com (C.S.); 2 Cardiology Department, Cardiovascular Diseases Institute “Prof. Dr. George I.M. Georgescu”, Iași 700503, Romania

**Keywords:** biomarkers, venous thrombosis, diagnosis, D-dimer, microparticles, E-selectin, P-selectin

## Abstract

Venous thrombosis is a common and potentially fatal disease, because of its high morbidity and mortality, especially in hospitalized patients. To establish the diagnosis of venous thrombosis, in the last years, a multi-modality approach that involves not only imaging modalities but also serology has been evolving. Multiple studies have demonstrated the use of some biomarkers, such as D-dimer, selectins, microparticles or inflammatory cytokines, for the diagnosis and treatment of venous thrombosis, but there is no single biomarker available to exclusively confirm the diagnosis of venous thrombosis. Considering the fact that there are some issues surrounding the management of patients with venous thrombosis and the duration of treatment, recent studies support the idea that these biomarkers may help guide the length of appropriate anticoagulation treatment, by identifying patients at high risk of recurrence. At the same time, biomarkers may help predict thrombus evolution, potentially identifying patients that would benefit from more aggressive therapies. This review focuses on classic and novel biomarkers currently under investigation, discussing their diagnostic performance and potential benefit in guiding the therapy for venous thrombosis.

## 1. Introduction

Venous thromboembolic disease (VTE) is still one of the major challenges in cardiovascular disease or Hematology because it remains a significant source of morbidity and mortality. It encompasses two major clinical entities: deep vein thrombosis (DVT) and pulmonary embolism (PE). Globally, it represents the third most frequent acute cardiovascular syndrome, behind myocardial infarction and stroke [1,2]. Annual incidence rates for venous thrombosis range from 104 to 183 per 100,000 populations in Europe, being higher in African Americans and lower in Asians [3]. In adults, the rates of VTE increase with age and data from cross-sectional studies show that the incidence of VTE is eight times higher in patients aged ≥ 80 years than in the fifth decade of life [4]. On the other hand, in sick children there is evidence of a difference in age of occurrence of VTE [1,4]. Venous thromboembolic disease persists as a source of major morbidity and mortality. Up to 10% of patients who survive after an initial episode of unprovoked venous thromboembolic disease will develop severe post-thrombotic syndrome, 30% will have a recurrent event within 10 years and up to 50% may develop aspects of post-thrombotic syndrome [5,6,7].

Considering the significant morbidity and mortality associated with venous thromboembolic disease, an accurate and timely diagnosis is definitely needed to initiate a proper treatment. Unfortunately, the nonspecific symptoms and lack of specific signs of venous thrombosis can lead to a delayed or inaccurate diagnosis, which can result in inferior patient outcomes [8,9]. Therefore, in front of a patient with a potential DVT it is very important to remember three things: some patients with symptoms of DVT may not have a clot, there is no available biomarker capable to exclude DVT in all patients [7] and history and physical exam alone are not confident to exclude the diagnosis of DVT [1,10]. 

Thus far, there has been no single serum marker available to exclusively confirm the diagnosis of VTE and the most widely used and accepted is D-dimer. It has a high sensitivity but a lack of specificity necessary to confirm the diagnosis, and therefore, is useful for the exclusion of the disease. This is the reason why additional studies including duplex ultrasound, echocardiography, computed tomography (CT) scans or pulmonary angiography are necessary for diagnosis [11]. In this context, ongoing research efforts target and support the utility of various plasma markers as novel ”biomarkers” for VTE including selectins, microparticles, interleukin-10 and other inflammatory markers. The aim of our review was to analyze the currently used biomarkers involved in the pathophysiology and diagnosis of venous thrombosis. They are very important not only in venous thrombogenesis but also to target the length of appropriate anticoagulation treatment and to predict thrombus biological activity [12]. 

## 2. D-Dimer

**Figure 1:**
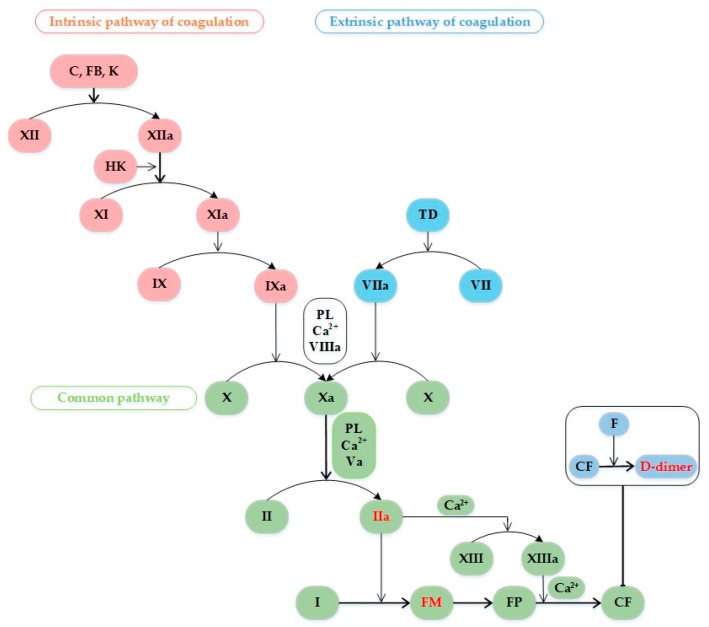
The extrinsic and intrinsic pathway of coagulation and the formation process of D-dimer. Abbreviations: Roman numerals, the clotting factors; C, collagen; FB, foreign body; K, kallikrein; HK, high-molecular-weight kininogen; PL, phospholipid; TD, tissue damage; FM, fibrin monomer; FP, fibrin polymer; CF, crosslinked fibrin; F, fibrinolysinum.

This is the most well-established biomarker of an ongoing fibrinolytic process. D-dimers are one of several fragments that are produced when plasmin cleaves fibrin, thus, they represent the expression of fibrin formation and degradation occurring during the fibrinolytic activity of clot breakdown [13]. Thrombin converts fibrinogen into soluble fibrin monomer, which then spontaneously polymerizes to form the soluble fibrin polymer. In the presence of calcium, thrombin also activates factor XIII, which crosslinks the fibrin polymer, producing cross-linked fibrin. Fibrinolysinum cleavage of the factor XIII activated-mediated cross-linked fibrin produces D-dimer [14] (Figure 1).

Over time, the role of D-dimer assays in clinical practice has evolved. Thus, they were first introduced in clinical medicine in the 1970s, when they were used to check for evidence of disseminated intravascular coagulation. These first-generation assays were more of a blunt tool than a fine-tuned diagnostic instrument because they detect both fibrinogen degradation products and fibrinogen. In time, there was an improvement in the performance of D-dimer assays and they are used not only in the algorithm to exclude acute thrombosis but also to predict which patients are at higher risk of recurrent thrombosis when anticoagulants are stopped [15].

There are more available tests for D-dimer quantification, but enzyme-linked immunosorbent assays (ELISA) methods are considered the reference test because they have a high sensitivity for low-levels of D-dimer [15]. The disadvantages, on the other hand, are represented by the fact that it is a time-consuming technique, with a turnaround time of 2–4 hours, which has a moderate specificity. The other available tests for D-dimer quantification are enzyme-linked immunofluorescence assay, latex-enhanced immunoturbidimetric and whole-blood assays, the latter having the advantage that it can be performed at the bedside, with a turnaround time of 2–5 minutes and a higher specificity, but the disadvantage of a lower sensitivity [15]. 

D-dimer testing gives a measure of ongoing fibrinolysis and has a highly negative predictive value, allowing the exclusion of an ongoing process of clot formation, which means that it can be used to exclude DVT in a patient with low pretest probability [16,17,18]. For example, only a highly sensitive D-dimer assay can exclude deep vein thrombosis in a moderate-risk patient, whereas a moderately sensitive D-dimer assay needs additional testing for the final diagnosis [19]. However, D-dimer assay has a high sensitivity but a low specificity. For the diagnosis of DVT, the sensitivity of D-dimer is 96%, but it has a low specificity (40%) and low positive predictive value (48%) [20,21,22]. Thus, it will miss few patients with venous thrombosis, but, due to low specificity, it will also give a great number of false-positive results that will lead to unnecessary paraclinical investigations [23]. In order to improve the utility of D-dimer measurement in patients with suspected venous thrombosis, it is usually combined with other tests. 

Despite its high sensitivity, D-dimer testing might prove less specific because increases in many situations, such as surgery, pregnancy, infection, inflammatory disorders, stroke, disseminated intravascular coagulation or cardiovascular disease [10,24,25,26,27,28,29,30,31] (Table 1). 

It is also very important to mention that D-dimer levels increase with age, thus, older patients may have a positive result even if they do not have a venous thrombosis [32]. This tendency of D-dimer to increase with age can increase the D-dimer’s specificity, reducing in this way the false positive results, without affecting the high sensitivity [33]. In patients over 50 years old, we may use an age-adjusted cut-off value, which is calculated by multiplying with 10 the age of the patient; thus, if the patient is 70 years old, the cut-off value for D-dimer would be 700 µg/mL [34,35]. 

Several prospective studies evaluated D-dimer levels for use in predicting the risk of VTE recurrence and guiding the length of therapy for initial VTE [36,37]. Cosmi et al. studied the risk of recurrence after anticoagulation withdrawal for a first idiopathic deep vein thrombosis, based on D-dimer levels in combination with residual venous obstruction by ultrasound, on the day of oral anticoagulation suspension. They included 400 patients and noted that abnormal D-dimer levels at one month after anticoagulation withdrawal are an independent risk factor for recurrent venous thrombosis (multivariate hazard ratio, HR of 3.32), while residual venous obstruction on duplex ultrasound on the day of oral anticoagulant suspension does not influence the rate of recurrence [36,37]. Another study that utilized D-dimer testing to determine the duration of anticoagulant therapy was PROLONG study, which included 608 patients with a first episode of unprovoked venous thromboembolism that were followed for a mean duration of 1.4 years after at least 3 months of therapy with warfarin [38]. The authors summarized that there is a clear benefit of prolonged anticoagulant therapy in patients with an abnormal level of D-dimer one month after the discontinuation of anticoagulation. 

When we talk about duration of anticoagulation in patients with unprovoked VTE, it is very important to weigh the benefit of treatment with the risk of bleeding, considering the fact that these patients are at high risk for recurrence if anticoagulant treatment is stopped. In order to stop safely anticoagulant therapy in patients with unprovoked VTE, several scales, that also use D-Dimer, including the HERDOO2 rule ( Hyperpigmentation, Edema or Redness in either leg; D-dimer level ≥ 250 µg/L; Obesity with body mass index ≥ 30; or Older age ≥ 65 years), DASH prediction score (abnormal D-dimer level at 1 month after stopping anticoagulation; Age < 50 years; male Sex; Hormonal therapy at VTE onset) and Vienna Prediction Model, have been proposed [39,40,41,42,43,44,45]. 

The HERDOO2 rule has been proposed as a guide to identify low-risk women with unprovoked venous thromboembolism who can safely stop anticoagulants after 5–12 months of anticoagulant treatment. Based on the presence of post-thrombotic signs, D-dimer level, body mass index and age, women receive a score from 0 to 4, and those with 2 or more should continue oral anticoagulants [39,40]. 

The DASH prediction model is used to identify patients at a low risk of recurrence of VTE. An abnormal D-dimer level at 1 month after stopping anticoagulation (D), age < 50 years (A) and male sex (S) increase the risk of recurrence with +2, +1 and +1, respectively, while estroprogestin use at the time of the index event reduces it (with a score of -2). Patients that have a score ≤ 1 are considered to be at “low risk” because their predicted annual VTE recurrence risk is < 5% [41,42]. 

Vienna Prediction Model allows prediction the risk of recurrent VTE at different time points after stopping anticoagulant therapy, using nomograms, in patients with unprovoked VTE. This risk can be estimated based on three parameters: sex, location of VTE (pulmonary embolism, proximal or distal deep vein thrombosis) and D-dimer level at 3 weeks after stopping anticoagulation [43,44,45].

According to these scores, male patients and those with elevated D-Dimer levels either during or up to one month after discontinuing therapeutic anticoagulation, have a high risk of recurrent VTE (> 5% per year). In these cases, there is a benefit of prolonged anticoagulant treatment [45].

## 3. P-Selectin

P-selectin is emerging as one of the novel biomarkers for venous thromboembolism, considering the fact that it is associated with vascular and thrombotic diseases. Together with L-selectin and E-selectin, it is a part of cell adhesion molecules [46,47,48]. It is found in the α-granules of platelets and the Weibel-Palade bodies of endothelial cells and during cell activation is redistributed onto the cell surface and partially released into the circulation in its soluble form (sP-selectin). Soluble P-selectin plays an important role in platelet and leukocyte adhesion to the activated vessel wall and mediates some of the effects of inflammation in thrombosis [7]. Even if they are present in endothelial cells, they are considered a reliable marker of in vivo platelet activation [49,50] because platelets are currently considered the major source of circulating sP-selectin in healthy patients [51,52]. Elevated P-selectin levels have been demonstrated in patients with deep vein thrombosis in several studies [21,53,54]. Comparing the levels of P-selectin in 21 patients with DVT vs. 30 healthy controls, Rectenwald et al. demonstrated elevated values in those with DVT (88.7 ng/mL vs. 54.5 ng/mL) [21]. This suggested the clinical applicability of P-selectin measuring to assess the risk of deep vein thrombosis. In another study, Papalambrosm et al. demonstrated a notable decrease in P-selectin levels after 7 days of therapeutic heparin therapy in patients with DVT [54]. 

Other studies evaluated the combination of sP-selectin with other measurements such as D-dimer, C-reactive protein (CRP), microparticles (MPs) and clinical Wells Score in confirming or excluding the diagnosis of DVT. Ramacciotti et al. used a logistic regression model to analyze a total of 178 patients presenting for US doppler imaging to diagnose DVT [55]. They included 62 patients with DVT and 116 patients without DVT and demonstrated that a value of sP-selectin > 90 ng/mL combined with a Wells Score ≥ 2, could establish the diagnosis of DVT with a specificity of 96% and a positive predictive value of 100%. On the other hand, a value of sP-selectin < 60 ng/mL combined with a Wells Score < 2, could exclude the DVT diagnosis with a specificity of 33%, a sensitivity of 99% and a negative predictive value of 96% [55]. In conclusion, the results of this study suggest that a combination of sP-selectin with Wells Score could confirm or exclude the diagnosis of DVT. 

Another similar study, which included 159 patients, demonstrated that patients with confirmed DVT had statistically significantly higher levels of P-selectin, D-dimer, CRP and von Willebrand factor [56]. In this study, sP-selectin levels > 90 ng/mL combined with a Wells Score ≥ 2, could establish the diagnosis of DVT with a specificity of 96%, a positive predictive value of 89% and a negative predictive value of only 77%, thus, sP-selectin levels are more effective in ruling–in the diagnosis of DVT than in excluding it. On the contrary, D-dimer measurements proved more effective than sP-selectin in excluding the diagnosis of DVT, as a value of D-dimer < 500 ng/mL combined with a Wells Score < 2 had a negative predictive value of 100% [56]. 

Studies on animal models demonstrated that inhibition of P-selectin can impact the development of atherosclerosis, fibrin deposition and thrombus growth [21,57,58,59]. In humans, high levels of sP-selectin are also found in cardiovascular disease, atrial fibrillation and diabetes [60], and may be predictive of future adverse cardiovascular events [21,61,62]. 

In conclusion, P-selectin may be one of the most promising novel biomarkers for venous thrombosis, considering the fact that it is a noninvasive test with a highly positive predictive value that can guide the initial management when imaging tests cannot be completed; additionally, in combination with other diagnostic tests, it offers a promising target for prospective management strategies. 

## 4. Microparticles

Microparticles are small membranous vesicles < 1 micron in size, released from the plasma membranes of platelets, leukocytes, red cells and endothelial cells in response to cell activation, injury or apoptosis [21,63,64,65]. Microparticles contain surface proteins responsible for their procoagulant activity, such as tissue factor and phosphatidylserine. Monocyte-derived microparticles activate coagulation especially via tissue factor [66,67], while platelet-derived MPs trigger thrombus propagation both by initiating thrombin generation independently of tissue factor and by exposing phosphatidylserine on their surface [67,68]. 

Microparticles are implicated in the development of several vascular, inflammatory and coagulation disease processes, and in particular cancer associated VTE [69,70]. Additionally, elevated levels of platelet-derived microparticles are associated with high risk angiographic coronary obstructive lesions and are considered a marker of platelet activation in cardiovascular disease [71,72]. In patients with VTE, studies demonstrated high levels of microparticles, microparticle-monocyte conjugates and an increased platelet activation, which supports the idea that they play a central role in both the coagulation and inflammatory processes during acute thrombogenesis [73]. Over time, there were discovered a lot of microparticles but it is difficult to use them as a diagnostic tool because there is no literature consensus on their measurement [73,74]. 

Considering that there is a poor standardization of analytical methods for microparticles detection [75], there is a discrepancy between the results of different studies and many of them linked elevated levels of microparticles with future occurrence of thrombosis [76,77], while others did not demonstrate their role as predictive biomarker [78,79].

For example, in order to determine different cutoff points for microparticles, D-dimer and P-selectin that could be used in early diagnosis and prediction of deep vein thrombosis in symptomatic patients with normal duplex ultrasound, Ghozlan et al. demonstrated that their concentration was significantly higher in duplex–positive patients [51]. Additionally, the use of either P-selectin or D-dimer alone or in combination did not show any improved sensitivity or specificity over microparticles when used alone [51]. The results of this study support the results of other studies published by Chirinos et al. and Bal et al. [73,80] who demonstrated elevated levels of microparticles in patients with deep vein thrombosis. Elevated levels of microparticles in thrombosis were also observed by Digant-George et al. [81] and Tesselaar et al. [79] in patients with antiphospholipid syndrome and cancer. 

On the other hand, the results of other studies [21,82] are in contradiction with those already presented. For example, Rectenwald et al. [21] and Ay et al. [82] observed that there was no statistically significant difference between microparticles levels in duplex-positive and duplex-negative patients. This difference could be explained by the methods used in measuring microparticles levels (as in flow cytometry, for example, some analytical conditions influence the results) and also by the type of specimen used for microparticles assay [51]. 

Other studies suggest that microparticles carry both nuclear and cytoplasmic ribonucleic acid (RNA) [83] and deoxyribonucleic acid (DNA) [84], especially microRNA. Additionally, microparticles have an important role in transferring microRNA from endothelial progenitor cells to endothelial cells, and this horizontal transfer of genetic mass between platelets and endothelial cells may influence thrombosis [14]. 

Although the implication of microparticles in venous thrombosis is fascinating, clinical research on microparticles is influenced by the variations in pre-analytical conditions of the available detection methods, that makes the results widely variable [73,85,86]. 

It is very important to identify the most efficient biomarker in discriminating patients without deep venous thrombosis from those with thrombosis, based on its sensitivity and specificity. Analyzing the results of the studies published thus far, we observed that only a few of the potentially biomarkers used for the diagnosis of DVT, have studies regarding their sensitivity and specificity (Table 2) [16,51]. D-dimer testing has the highest sensitivity and until now is the only biomarker used in routine clinical practice, but there are also good expectations from the others. 

## 5. E-Selectin

E-selectin follows P-selectin up-regulation and studies in a murine model of venous thrombosis demonstrated that it amplifies the effects of P-selectin and the thrombotic response [87,88]. The association of Ser128Arg with recurrent venous thromboembolism in humans was studied by Jilma et al. [89]. In this study, the authors demonstrated that homozygosity for Ser128Arg mutation increases the probability for recurrent thrombosis and was considered an independent predictor of recurrent venous thrombosis compared to heterozygous population (HR 4.1; 95% CI, 1.5–11.4 vs. HR 1.1; 95% CI, 0.6–1.9) [89]. In different studies E-selectin polymorphism was associated with tissue factor-mediated coagulation, atherosclerosis, myocardial infarction and restenosis after transluminal percutaneous coronary angioplasty and may be associated with recurrent venous thrombosis [89,90,91,92,93]. Coleman et al. demonstrated that inhibition of both E-selectin and P-selectin is an effective strategy to reduce venous thrombosis [12].

## 6. Thrombin

Thrombin has an important role in the acceleration of the coagulation cascade and in clot formation [94]. Thrombin can be efficiently measured by immunologic-based assays in patients who are suffering the activated coagulation cascade caused by tissue factor and phospholipids [95]. Thrombin generation can be expressed in different ways: the lag time (time until thrombin burst appears), the peak thrombin generation (the maximal concentration of thrombin formed at a given point in time) and the endogenous thrombin potential (the area under the curve) [14]. 

Increasing evidence suggests a role for thrombin in VTE [96] and can also be useful as a predictive marker for thrombosis [97]. Increased levels of thrombin generation were observed in patients at risk of VTE [98] and also in patients with a prior history of VTE, and they were significantly correlated with FVIII level [99]. In a post-hoc analysis of a prospective study Lutsey et al. found that elevated basal peak thrombin was associated with the risk of recurrent venous thrombosis, independently of other VTE risk factors [100]. 

The implications of thrombin generation assay in hemostasis was assessed by Segers et al. [101]. The results of their study showed that the thrombin generation assay is sensitive to genetic variation in hemostasis-related genes and this makes it a promising instrument to identify novel genetic risk factors for venous thrombosis [101]. 

Considering the fact that a normal D-dimer level can exclude deep vein thrombosis in patients, it is possible that the combination of D-dimer and thrombin generation can increase the specificity, sensitivity and positive and negative predictive value for diagnosing or excluding venous thrombosis [14].

## 7. Factor VIII

During the coagulation cascade, there is an elevation in the pro-coagulant proteins, in particular the coagulation factor VIII (FVIII) [102,103]. Activated FVIII plays an important role in the activation of the common pathway of the coagulation cascade and in the thrombin generation from prothrombin. The Leiden Thrombophilia Study and VTE [103] was the first study who pointed out the association between high levels of FVIII and the risk of venous thrombosis. This hypothesis was subsequently confirmed by other studies [102,104,105,106,107,108,109], who also showed that elevated FVIII levels are associated not only with the diagnosis but also with the patient’s prognosis [103]. For example, Koster et al. evaluated, in a patient-control study, the role of the ABO blood group, von Willebrand factor and FVIII in the pathophysiology of deep vein thrombosis [103]. They observed that blood group, von Willebrand factor are involved in a common causal pathway of thrombogenesis, and their effects are mediated by FVIII, while FVIII is a risk factor in multivariate analysis. 

Evaluating the relationship between high plasma levels of FVIII and the risk of recurrent venous thrombosis, Kyrle et al. [107] observed that this relation was nonlinear, and the risk of recurrent thrombosis for patients with a FVIII level above 90^th^ percentile was 7.4 times higher than individuals with lower levels. They also highlighted that a high level of FVIII was a cause rather than a consequence of venous thrombosis, considering the fact that high levels of FVIII persist over time [110,111]. At the same time, Kraaijenhagen et al. observed that for each 10 UI/dl increased in FVIII, the risk of first VTE increased by 10%, and the risk of recurrent VTE increased by 24% [35]. 

Current research aims to identify the relation between venous thrombosis and FVIII in the genetic aspect, although only few new mutations or polymorphisms related to FVIII have been identified thus far [112,113]. 

## 8. Fibrin Monomer

Coagulation cascade involves a lot of processes such as platelet activation, increased activity of coagulant system and fibrin formation [14]. Fibrin monomers are produced by thrombin-mediated cleavage of fibrinogen and Vogel et al. assessed them as markers of intravascular activation of the coagulation system resulting in disseminated intravascular coagulation or deep venous thrombosis [114]. Schutgens et al. [115] suggested that fibrin monomers can be a valuable diagnostic tool for the early diagnosis of postoperative deep vein thrombosis, with a positive predictive value of 63%–73%, and they may aid in the diagnosis when combined with ultrasound examination and D-dimer assays [115]. Vogel et al. agree to this opinion and they found that fibrin monomer assay had a specificity of 73.2% and a sensitivity of 91.7% for the early diagnosis of postoperative deep venous thrombosis [116]. 

Even if both D-dimer and fibrin monomer are fibrin-related markers, there is an important difference between them: while D-dimer is a fibrinolysinum-mediated breakdown product of crosslinked fibrin in the post-thrombotic state [117], fibrin monomer results from thrombin-induced proteolysis of fibrinogen in a hypercoagulable state [94]. Thus, D-dimer could be used as a post-thrombotic marker, while fibrin monomer as a pre-thrombotic marker [118]. Therefore, evaluation, at the same time, of fibrin monomer and D-dimer levels may rise the sensitivity for the diagnosis of DVT [118]. 

## 9. Inflammatory Cytokines

More and more studies emphasize the role of inflammatory markers such as C-reactive protein and interleukin (IL)-1β, 6,8,10 in venous thrombosis. By influencing the expression of tissue factor, inflammatory cytokines provide a trigger that may lead to thrombotic disease [119]. Lab studies demonstrated that increased CRP levels had an important role in appearance of venous thrombosis [120,121]. There are also other studies that do not sustain this idea. For example, Tsai et al., in a prospective study, found that there is no relationship between plasma CRP level and development of venous thrombosis [104]. At the same time, Fox et al. using data from four studies that evaluated the role of CRP in the diagnosis of VTE, concluded that the sensitivity of CRP is 77% and the specificity is 66%, and plasma CRP level, used alone, is not useful to diagnose deep venous thrombosis [122]. 

High-sensitivity C-reactive protein levels may also be used to predict the risk of venous thromboembolism recurrence after discontinuation of anticoagulant treatment, in patients with cancer-associated thrombosis. Jara-Palomares et al. evaluated the D-dimer and high-sensitivity CRP levels after the withdrawal of anticoagulation, to predict the risk of VTE recurrence in patients with cancer-associated thrombosis [123]. The D-dimer and high-sensitivity CRP levels were determined when patients stopped the anticoagulant therapy and 21 days later. Patients were followed up for 6 months and both D-dimer and high-sensitivity CRP levels, after 21 days, were associated with VTE recurrence (HR 5.81; 95% CI: 1.1-31.7 for D-dimer, respectively, HR 9.82; 95 % CI: 19-52 for high-sensitivity CRP). The results of this study show that CRP and D-dimer may be potential biomarkers of VTE recurrence after withdrawal of anticoagulation in patients with cancer-associated thrombosis [123].

Association between VTE and other markers of inflammation such as tumor necrosis factor (TNF)-α, IL-1β, IL-6, IL-8, IL-10 and IL-12p70 was evaluated in a case–control study by Reitsma and Rosendaal [124]. The results of this study show that levels of TNF-α, IL-6 and IL-8 are risk determinants for venous thrombosis; the association was weak with IL-1β levels; there was no association for IL-12p70; the risk was decreased for the anti-inflammatory cytokine IL-10. These results are also supported by the study of Poredos et al. [125]. They found increased levels of IL-6 and IL-8 and decreased levels of IL-10 in patients with idiopathic venous thrombosis. Another important result of their study is the relation between endothelial dysfunction and the risk for VTE; thus, the thrombus was generated by involving two mechanisms: endothelial dysfunction and coagulation cascade.

Evaluating the role of IL-10 in thrombus formation, Downing et al. observed that neutralization of IL-10 increased thrombosis and inflammation, while supplementation with exogenous IL-10 decreased the inflammation and thrombus formation; therefore, IL-10 may be used as a therapeutic agent in the treatment of VTE [126]. In another case-control study, the authors observed that IL-10 G13 allele was an independent risk factor for VTE and IL-10 G10 allele may be an independent risk factor not only for venous thrombosis but also for recurrent disease, considering the fact that it was more frequent in recurrent disease [127,128]. 

Although all of these biomarkers mentioned above theoretically have connection with the pathobiology of deep vein thrombosis, each of them has a different role in diagnostic or prognostic purposes of DVT (Table 3).

## 10. Other Biomarkers

Many other biomarkers have been explored or are part of different trials, for the diagnosis of deep vein thrombosis or to predict the appearance of venous thrombosis [20]. 

Serum albumin, a negative acute phase reactant, is one of the biomarkers proposed for VTE risk [129,130], which increased continuously with decreasing levels of albumin [129]. 

Glycemic index before chemotherapy may also allow for venous thrombosis risk stratification in breast [131] and gastrointestinal neoplasia [132], independently of other well-known risk factors. This may be explained by the fact that in healthy nondiabetic subjects, increased blood glucose levels enhance blood coagulation [133]. 

Blood lipids are also markers for VTE onset in cancer out patients receiving chemotherapy [134]. Ferroni et al. demonstrated that patients with low high-density lipoprotein cholesterol (HDL-C) levels before chemotherapy, had a three-fold higher risk of venous thrombosis, independently of body mass index [134]. This was also confirmed by other studies that have shown that patients under statins had a lower risk of VTE than patients without this drug [135,136], possible as a result of statin’s capacity to modify endothelial function and to decrease the inflammatory response. 

Many of the current research aims to identify novel biomarkers that may be useful not only for the diagnosis and management of deep venous thrombosis but also for a better understanding of the pathophysiology of this disease.

Thrombin-antithrombin III complex (TAT) is induced by thrombin and is being extensively studied as an important and sensitive biomarker for venous thromboembolism. Studies indicate that TAT levels can be used as a screening procedure for venous thromboembolism after hip or knee replacement surgery [137,138] and also in patients with lower limb and pelvic fractures [139]. A recent study showed that thrombin-antithrombin complex may also be used to identify critically ill children who are at high risk of venous thromboembolism [140]. Tala et al. observed that higher levels of thrombin-antithrombin complex (Odds Ratio (OR): 31.54; 95% CI: 2.09–475.92) and lower levels of factor XIII (OR: 0.03; 95% CI: 0.002-0.44), but not prothrombin fragment 1+2, are associated with deep vein thrombosis [140]. In another recent study, Memon et al. evaluated the role of 92 circulating protein potentially involved in inflammation and cardiovascular disease, in order to identify new diagnostic biomarkers for deep venous thrombosis [141]. From the total of 30 proteins whose plasma levels were significantly different between DVT and non-DVT patients, after Bonferroni correction, plasma levels of seven proteins remained significantly different: P-selectin, transferrin receptor protein 1, von Willebrand factor, tissue factor pathway inhibitor, osteopontin, bleomycin hydrolase and ST2 protein. All of these seven proteins were significantly correlated with markers of hypercoagulability and they may become novel diagnostic biomarkers for deep venous thrombosis [141]. 

Other recent biomarkers have been or are currently explored for the diagnosis or venous thrombosis and they may be grouped into those with limited or no signal, mixed results or strong results, but they require further validation before considering their use in clinical practice [7,142,143] (Table 4).

Although many of these biomarkers are very promising, currently, they cannot be used to make decisions in the management of patients with venous thromboembolism because they do not have an established cut-off point in order to validate them, and most of them have not been used in clinical trials. Nowadays, in order to confirm the venous thromboembolism, a positive D-dimer test always needs to be associated with a positive radiological examination, such as ultrasound. In the absence of an objective method, currently, there are no further biomarkers available that can confirm the diagnosis of venous thromboembolism.

## 11. Conclusions

Classic and novel laboratory biomarkers are very important not only in the diagnosis of venous thrombosis but also in the management strategy. Currently, only D-dimer levels are used in routine clinical practice to make decisions regarding the management of venous thrombosis, but there are good expectations with novel biomarkers that are currently being studied. Ongoing data support the use of biomarkers to predict recurrence risk and guide length and modality of treatment for patients with venous thrombosis. Thus, the inclusion of molecular markers may potentially identify patients at high risk for venous thrombosis and recurrence that may need more aggressive anticoagulant treatment. 

## Figures and Tables

**Table 1:** Conditions for which the D-dimer may not be useful.

Patient Groups
Advanced age
Hospitalized patients
Burns
Cancer
Infection (inflammatory state or hemostatic disorders)
Postoperative (orthopedic or neurologic)
Pregnancy
Liver disease
Massive bleeding
Multiple traumatic injuries

**Table 2:** Sensitivity, specificity and predictive cut-off values of D-dimer, P-selectin and microparticles for the diagnosis of DVT.

Variables	Cut-Off Values	Sensitivity (100%)	Specificity (100%)
D-dimer (mg/L)	2.81	57	97
P-selectin (%)	30.2	54	94
Microparticles (mmol/L)	26	43	100

**Table 3:** The role of different biomarkers in the pathophysiology of deep vein thrombosis.

Biomarkers	Role in the Pathophysiology of DVT
D-dimer	Fibrin degradation products, formed when cross-linked fibrin is lysed by plasmin. They indicate the activation of thrombin and plasmin.
P-selectin	Cell adhesion molecule present in platelets and endothelial cells. Mediates the binding of platelets and endothelial cells with leukocytes, the transfer of tissue factor to platelets and triggers formation of leukocyte derived microparticles.
Microparticles	Small membranous vesicles, released from the plasma membranes of platelets, leukocytes, red cells and endothelial cells. Play an important role in the initiation and propagation of VTE through the development of their own procoagulant properties, enhancing intercellular communication and promoting inflammation.
E-selectin	Expressed by endothelial cells is associated with tissue factor-mediated coagulation.
Thrombin generation	A breakdown product of prothrombin, activates the platelets, factor V, VIII and converts fibrinogen to fibrin.
Factor VIII	Produced in liver sinusoidal cells and endothelial cells outside the liver, is a cofactor for factor IXa, which, in the presence of Ca^2+^ and phospholipids, converts factor X to the activated form.
Fibrin monomer	Results from thrombin-induced proteolysis of fibrinogen in a hypercoagulable state, being a pre-thrombotic marker.
Inflammatory cytokines	Released by leucocytes, endothelial cells, fibroblasts and other cell types that promote inflammation, they influence endothelial function and the expression of tissue factor.
Thrombin-antithrombin III complex	Is a good measure for thrombin level in the blood because it is formed as a result of the high level of thrombin caused by coagulation.
Prothrombin fragment 1+2	When prothrombin is converted into thrombin by factor Xa, their plasma levels are elevated.

**Table 4:** Potential biomarkers for the diagnosis of DVT.

Moderate to Strong Signal	Mixed Results	No/Limited Signal
Thrombin-antithrombin complex D-dimer Fibrinogen Hepatocyte growth factor Protein C Protein S Soluble P-Selectin Serum albumin Vascular cell adhesion molecule 1	C-reactive protein IL-6	Basic fibroblast growth factor CD40 ligand Chemokine ligands 2,3,4,5,8,11 Endocan E-selectin Granulocyte colony stimulating factor Interferon-γ IL 1-α, IL 1-β, IL-2, IL-4, IL-5, IL-7, IL-8, IL-10, IL-12, IL-13, IL-17, IL-22 L-arginine Matrix metalloprotease 1 Prothrombin fragment 1+2 Thrombopoietin Tumor necrosis factor α Vascular endothelial growth factor

IL, interleukine; CD, cluster of differentiation.

## Abbreviations

VTE	Venous thromboembolic disease
DVT	Deep vein thrombosis
PE	Pulmonary embolism
CT	Computed tomography
ELISA	Enzyme-linked immunosorbent assays
ELFA	Enzyme-linked immunofluorescence assay
CRP	C-reactive protein
MPs	Microparticles
PPV	Positive predictive value
NPV	Negative predictive value
RNA	Ribonucleic acid
DNA	Deoxyribonucleic acid
IL	Interleukin
TNF	Tumor necrosis factor
HDL-C	high-density lipoprotein cholesterol
TAT	Thrombin-antithrombin complex
CD	Cluster of differentiation
